# Chimpanzees process structural isomorphisms across sensory modalities

**DOI:** 10.1016/j.cognition.2017.01.005

**Published:** 2017-04

**Authors:** Andrea Ravignani, Ruth Sonnweber

**Affiliations:** a AI Lab, Vrije Universiteit Brussel, Brussels 1050, Belgium; b Department of Cognitive Biology, University of Vienna, Vienna 1090, Austria; c Language and Cognition Department, Max Planck Institute for Psycholinguistics, Nijmegen 6525, The Netherlands; d Department of Primatology, Max Planck Institute for Evolutionary Anthropology, Leipzig 04103, Germany

**Keywords:** Cross-modal, Matching, Analogy, Audio-visual, Touchscreen, Pattern perception

## Abstract

Evolution has shaped animal brains to detect sensory regularities in environmental stimuli. In addition, many species map one-dimensional quantities across sensory modalities, such as conspecific faces to voices, or high-pitched sounds to bright light. If basic patterns like repetitions and identities are frequently perceived in different sensory modalities, it could be advantageous to detect cross-modal isomorphisms, i.e. develop modality-independent representations of structural features, exploitable in visual, tactile, and auditory processing. While cross-modal mappings are common in the animal kingdom, the ability to map similar (isomorphic) structures across domains has been demonstrated in humans but no other animals. We tested cross-modal isomorphisms in two chimpanzees (*Pan troglodytes*). Individuals were previously trained to choose structurally ‘symmetric’ image sequences (two identical geometrical shapes separated by a different shape) presented beside ‘edge’ sequences (two identical shapes preceded or followed by a different one). Here, with no additional training, the choice between symmetric and edge visual sequences was preceded by playback of three concatenated sounds, which could be symmetric (mimicking the symmetric structure of reinforced images) or edge. The chimpanzees spontaneously detected a visual-auditory isomorphism. Response latencies in choosing symmetric sequences were shorter when presented with (structurally isomorphic) symmetric, rather than edge, sound triplets: The auditory stimuli interfered, based on their structural properties, with processing of the learnt visual rule. Crucially, the animals had neither been exposed to the acoustic sequences before the experiment, nor were they trained to associate sounds to images. Our result provides the first evidence of structure processing across modalities in a non-human species. It suggests that basic cross-modal abstraction capacities transcend linguistic abilities and might involve evolutionary ancient neural mechanisms.

## Highlights

• Chimpanzees had learnt to choose structurally symmetric patterns on a touchscreen.
• Playback of asymmetric sounds increased latency to choose symmetric visual patterns.
• Chimpanzees form cross-modal isomorphisms between visual and acoustic structures.
• Untrained skills for structural analogies can arise spontaneously in nonhuman animals.

## Graphical abstract

**Figure:**
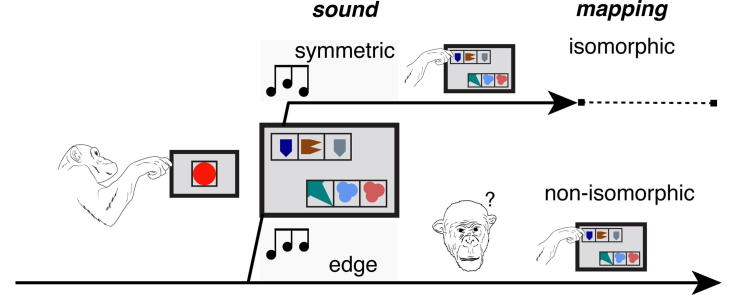

## 1 Introduction

**Fig. 1:**
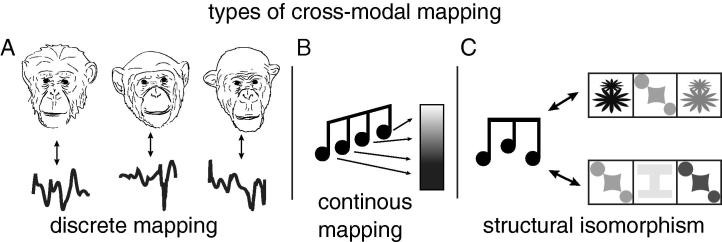
Types of cross-modal correspondences. Cross-modal mappings can be discrete (A), continuous (B), or isomorphic, involving whole structures mapped across domains (C), crucially with no reliance on previous specific associations between constituent elements (the diagonal symbol is successfully associated to both the high and low note).

Different forms of cross-modal processing exist in nature. A *discrete mapping* is a pair-wise association between distinct units in different domains (Fig.1A), for instance mapping faces to voices (Jordan, Brannon, Logothetis, & Ghazanfar, 2005). Apart from humans, some animal species form such cross-modal representations of conspecifics, as shown for monkeys (Adachi and Fujita, 2007, Adachi and Hampton, 2011, Sliwa et al., 2011), chimpanzees (Izumi and Kojima, 2004, Martinez and Matsuzawa, 2009), dogs (Adachi, Kuwahata, & Fujita, 2007), and horses (Proops et al., 2009, Seyfarth and Cheney, 2009). After learning to associate specific tones to specific colours, tones alone are enough to selectively activate colour neurons in primates’ neocortex (Fuster, Bodner, & Kroger, 2000). Also fruit flies exposed to combinations of visual and olfactory stimuli develop a cross-modal memory, which can be retrieved by light or odour alone (Guo & Guo, 2005) with no need of a neocortex. The diffusion of discrete mappings suggests they can, though need not, build upon basic neural mechanisms seemingly available to a range of organisms (Carew, 2000).

A *continuous mapping* relates graded percepts across modalities (Fig.1B), e.g., when deeper voices are associated to larger body sizes (Ghazanfar et al., 2007). Human infants spontaneously map more intense sound to brighter light (Lewkowicz & Turkewitz, 1980). Chimpanzees also show a similar sort of graded mapping spontaneously: When trained to discriminate light from dark squares, they perform better when white is suddenly paired with a high-pitched sound and black with a low-pitched sound than vice versa (Ludwig, Adachi, & Matsuzawa, 2011). Some continuous mappings have been hypothesized to be innate, synesthetic-like associations, possibly necessary for the evolution of human language via bootstrapping of sound-form or sound-gesture pairs (Cuskley and Kirby, 2013, Hubbard and Ramachandran, 2003, Ramachandran and Hubbard, 2001).

Finally, cross-modal *isomorphisms* require recognition that two percepts in different modalities share a common *structural* property. Isomorphisms combine the discreteness (and possible arbitrariness) of discrete mappings with structural features, partially found in continuous mappings as well. The three-note sequence in Fig.1C is isomorphic to both visual sequences; in particular, all sequences are structurally symmetric, i.e. they begin and end with the same (note or shape) element, with a different element between. This similarity transcends the particular physical characteristics of the stimuli and cannot be obtained by simply combining discrete mappings: isomorphisms instead map similar structures across modalities. Indeed, humans exposed to a visual sequence (e.g., nonsense strings of letters where H always occurs between two Ls) can tell whether unfamiliar sound sequences contain similar structural regularities (high-pitched sounds always occur between two low-pitched sounds) (Altmann et al., 1995, Conway and Christiansen, 2006).

Processing analogies requires understanding same/different identities as well as relations between relations among items composing a stimulus. This cognitive processing ability is often tested using a relational-matching-to-sample (RMTS) paradigm (e.g., Cook and Wasserman, 2007, Fagot and Parron, 2010, Fagot et al., 2001): here a subject has to match a sample (AA) to a test stimulus (BB) with properties analogous to the sample, while rejecting a non-analogous stimulus (bb or BX). To identify *structural* analogies in patterns, an item-independent representation of a structural rule (e.g., XYX is analogous to ABA and XXY is analogous to AAB) has to be formed (Spierings & ten Cate, 2016). As similar patterns of regularities exist in different modalities some isomorphisms transcending sensory categories are straightforward for humans. The reader is, for instance, establishing an isomorphism when interpreting a visual representation of a low-high-low note triplet (Fig.1C) as a low-high-low sound. Crucially, this is not amenable to mapping specific sounds with specific visual configurations (except for those few humans with absolute pitch), but to mapping one low-high-low *structure* in vision to another in audition (thus forming an analogy between the structure of a visual and an acoustic stimulus).

Humans are capable of both cross-modal mappings and cognitive isomorphisms. Like humans, other animals’ brains have been shaped by evolution to detect and take advantage of structural properties in environmental stimuli, e.g., social information, such as rank hierarchies and kin relations, or ecological information, such as fruiting patterns of trees (Seyfarth and Cheney, 2009, Sonnweber et al., 2015). Indeed, many animal species can learn experimentally-generated statistical and structural patterns within one modality (ten Cate, 2014, ten Cate and Okanoya, 2012). Although non-human animals are capable of modality-specific structure learning, discrete/continuous cross-modal mappings, and even second order relational matching (Fagot and Parron, 2010, Smirnova et al., 2015) to date cross-modal structural isomorphisms have only been shown in humans and computer-simulated neural networks (Dienes, Altmann, and Gao, 1995, Dienes, Altmann, Gao, and Goode, 1995, Hauser and Watumull, 2016).

**Fig. 2:**
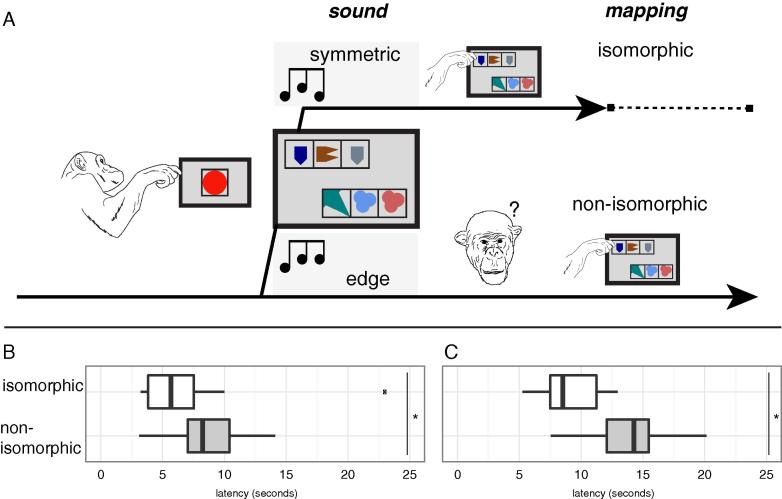
(A, left) Schematic representation of one trial. Trials always started with the presentation of a red circle: once the chimpanzee touched it, the sound triplet was played, the two visual sequences shown and chimpanzees’ latency to respond recorded. Boxplots of FK’s (B) and KL’s (C) latencies in providing the correct response. Median latencies across trials were significantly shorter (see main text and Table 1) in the isomorphic than in the non-isomorphic condition, namely 5.68 vs. 8.25 s (ape FK) and 8.52 vs. 14.27 s (KL).

Here we show for the first time that two non-human individuals can map isomorphic structures across modalities. Inconsistencies across modalities result in longer latencies to respond (Gallace and Spence, 2006, Miller, 1991). Hence, if a structural regularity is shared between modalities, or encoded on a modality-general level, input in one modality (e.g., auditory) should influence response latencies to stimuli in another modality (e.g., visual), depending on whether the structure of a stimulus in one modality is equivalent to (isomorphic to) or inconsistent with (non-isomorphic to) the stimulus structure in another modality. Thus we hypothesized that if chimpanzees perceived visual and auditory symmetric triplets as isomorphic, presentation of symmetric or edge auditory triplets would differentially affect processing of the symmetric visual triplet. Consequently, an inconsistent audio-visual pairing (Fig.2A, bottom timeline) should increase the time needed to respond (Gallace and Spence, 2006, Ludwig et al., 2011) relative to an isomorphic audio-visual pairing (Fig. 2A, top timeline).

## 2 Materials and methods

Two chimpanzees (FK, male, and KL, female, both 20 years of age) from Budongo Trail, Edinburgh Zoo (Ravignani et al., 2013) participated in this study. Chimpanzees lived socially with conspecifics in outdoor and indoor enclosures. Food was provided between four to five times per day while water was available ad libitum. During training and experiments, individuals could leave the sessions at any time and were not separated from their social group. Every time one individual participated in the experiment, a keeper distracted other individuals with husbandry training (Sonnweber et al., 2015).

The board of the Living Links - Budongo Research Consortium (Royal Zoological Society of Scotland) and the ethics board of Life Sciences, University of Vienna (approval number: 2014-010) approved this research. Only positive reinforcement techniques, and no invasive methods, were used. Procedures complied with Austrian, British, and EU legislations.

Chimpanzees had been previously trained to reliably choose visual sequences with identical shapes as first and last elements (constituting a dependency rule between these elements; Sonnweber et al., 2015) in a two-alternative-forced choice (2AFC) task. After the training phase, individuals were (i) tested for generalization abilities (coloration of shapes, novel shapes, and stimulus length) and (ii) presented with visual foil stimuli (positions and repetitions of dependent elements). Both chimpanzees mastered the generalization tests and were sensitive to the positional relation between dependent elements.

For testing cross-modal isomorphism processing, triplets of shapes or sounds served as stimuli. Triplets were chosen as the simplest testable pattern containing structure (Chen, van Rossum, & ten Cate, 2014). In a 2AFC task, chimpanzees were presented with pairs of visual patterns on a touch-sensitive screen and could respond by touching one of them (Sonnweber et al., 2015). The two triplets (Fig.2A, large screen), each composed of 3 horizontally arrayed, black-framed geometrical shapes in different colours, were: (i) one ‘symmetric’ triplet, consisting of two identical geometrical shapes separated by a different shape, which was positively reinforced in previous experiments (Sonnweber et al., 2015), and (ii) one ‘edge’ triplet, consisting of two identical geometrical shapes either followed or preceded by a different shape (never positively reinforced). Each geometrical element composing a visual pattern could have any of seven colours and thirty shapes (analogous to the visual stimuli used in Sonnweber et al., 2015). Visual sequences (all tokens presented simultaneously) occurred after one of two sound sequences was played: (i) a symmetric triplet, containing two high tones separated by a low tone (or vice versa) and isomorphic to the structure of symmetric images, or (ii) an edge triplet, where two consecutive high (or low) tones, were preceded or followed by a low (or high tone). Triplets were concatenated pure sine wave tones (detailed methods: Ravignani, Sonnweber, Stobbe, & Fitch, 2013). All stimuli lasted one second and contained three tones of 300 ms each, separated by 50 ms silence. The sounds were randomly sampled from low (200 ± 4 Hz) and high (400 ± 16 Hz) tone categories. Within-category variability in sounds and shapes was introduced so the animals could focus on categorical properties, rather than individual element features (Ravignani, Sonnweber, et al., 2013, Ravignani et al., 2015, Sonnweber et al., 2015, ten Cate and Okanoya, 2012, van Heijningen et al., 2009).

Crucially, visual and auditory stimuli used for the same categories could have any shape or frequency, as long as both ‘same’ stimuli in a pattern belonged to the same tone or shape category. The same held for the “different” category. Hence, the two ‘different’ geometrical shapes in a pattern (e.g., a triangle and a square) were mapped to tones from different tone categories (e.g., a high and a low tone, or vice versa), and all elements used as ‘different’ could also be used as ‘same’ in other trials (e.g., two adjacent triangles or squares mapped to two adjacent high or low tones). Any two same shapes could correspond to any two tones sampled from the same tone category and any two different shapes could be mapped to any two tones sampled from different tone categories. Stimuli were produced and data was analyzed using custom-written scripts in Python 2.7 and SPSS19 (Ravignani, Sonnweber, et al., 2013, Ravignani et al., 2015, Sonnweber et al., 2015).

We tested whether a structure (such as the symmetric arrangement) learned in the visual domain was available to other domains using a *cross-modal interference* paradigm. The chimpanzees did not receive any training for this experiment other than the previous, purely visual training to choose symmetrical patterns (Sonnweber et al., 2015). Test trials (which were not fed-back or rewarded) started with a screen displaying a red circle (and were preceded by reinforced pre-trials see Figs. 2 and S2 in Supplement). When the individual touched the circle a sound triplet was played, either isomorphic (symmetric; first and last tone matched) or non-isomorphic (edge; first and last tone differed) with the symmetry rule reinforced and learned in the visual domain. Immediately after the acoustic sequence ended (as temporal proximity between two stimuli increases the likelihood of multimodal integration; Spence, 2011), two visual triplets, one symmetrical, one with same element repetitions at the edge, were displayed until the individual touched either of them. Latencies to respond from the onset of a trial (i.e. presentation of the red circle) were measured. To avoid a drop of motivation every test trial was preceded by a pre-trial, where the correct choice of a red circle over a green one was rewarded (see also supplementary material). A one-second inter-trial-interval was embedded between test and pre-trials.

Test stimuli were sampled from random acoustic-visual stimuli combinations, 50% isomorphic (symmetric acoustic pattern matching the visual rule) and 50% non-isomorphic (edge acoustic pattern violating the symmetric visual rule) acoustic-visual pairings (see Table S1 in Supplement). Chimpanzees were tested on a strict voluntary basis until the end of our agreement to use the research premises. Chimpanzee KL underwent 20 isomorphic and 19 non-isomorphic trials. Chimpanzee FK underwent 38 isomorphic and 39 non-isomorphic trials. Inclusion (see Supplement) or exclusion (to obtain a balanced sample, see Section 3.) of FK’s last non-isomorphic trial and KL’s last isomorphic trial leaves significance of all statistical tests and our conclusions unchanged.

## 3 Results and discussion: Isomorphic sounds shorten latencies to choose correct visual patterns

Both chimpanzees were significantly slower in choosing the correct symmetric visual triplet after hearing an edge sound triplet rather than an isomorphic symmetric triplet (Mann-Whitney U test on correct trials; chimpanzee FK: U(33) *=* 80, Z = −2.384, p = 0.017, see Fig.2B; chimpanzee KL: U(15) = 10, Z = −2.440, p = 0.014, see Fig.2C). As these acoustic sequences were completely novel to the animals before the experiment, their structural properties must have interfered with processing of the learnt symmetry rule.

Chimpanzees were never trained to associate specific sounds with images; hence simple associative learning cannot explain our results (cf. Berwick, 2016). Edge stimuli have the same proportion of element types as symmetric stimuli: simple counting the number of element types or comparison of entropy across modalities are insufficient alternative explanations (Ravignani et al., 2015, ten Cate et al., 2010, van Heijningen et al., 2009).

One might argue that the observed results might also have occurred if individuals simply reacted differently to the two types of auditory stimuli without even perceiving the visual patterns (e.g., hesitating to react after hearing edge sound sequences as opposed to symmetric sound sequences). If auditory stimulus type alone affected response latency, we would observe different latencies between conditions also in trials where chimpanzees chose the visual edge (negative) stimulus. This however was not the case. Latencies did not differ between auditory conditions when visual edge triplets were chosen (Mann-Whitney U test, individual FK: N = 41, U(39) = 199, W = 452, Z = −0.261, p = 0.806; individual KL: N = 21, U(19) = 48, W = 126, Z = −0.426, p = 0.702).

Success in the reinforced pre-trial seems to partially explain latencies (Table 1). Across conditions and individuals, 5 of the 8 possible correlations are positive and significant: hence, success in a pre-trial might induce longer latencies. However, these significant correlations are spread quite unsystematically across conditions, suggesting that reinforcement in the pre-trials might contribute to, although it is not the only factor responsible of, our main result.

Error rates were extremely high, probably due to sudden change in the experimental procedure (type of pre-trials, introduction of sound files played), but comparable across priming conditions (FK: 50% vs. 56%; KL: 50% vs. 63%). Choosing the visual edge stimulus represents a failure in the trial, attributable to several potential factors (e.g., lack of concentration, distractions). Therefore we would not expect a difference in latencies to respond depending on the structure of the acoustic stimulus in failed trials. Future studies should provide a better control condition, playing the same sound triplets for visual stimuli on which isomorphism can and cannot be mapped onto.

Finally, both subjects showed no significant association between auditory stimulus heard and visual stimulus chosen (Fisher’s exact test: p = 0.650 for ape FK and p = 0.523 for ape KL). In other words, edge sounds did not persuade chimpanzees to choose the wrong visual triplet. This could be expected, as chimpanzees were *never trained* to *match* similar structures across modalities. Even though the Fisher exact test did not reach significance, it is interesting to notice how matched audio-visual pairs are the most frequent combinations in each individual’s contingency table (diagonal entries in bold in Table 1). A perfect, spontaneous audio-visual match to sample would result in contingency tables being purely diagonal. This suggests that the chimpanzees might have spontaneously shifted their choice towards the congruent asymmetric visual stimuli after hearing “edge” sound.

## 4 General discussion and conclusions

Our results provide the first evidence that two non-human animals have sensory binding capacities beyond discrete/continuous mappings. Moreover, our experiment introduces a successful, though simple paradigm useful to test additional individuals and species. We were only able to test two chimpanzees, employing the simplest imaginable structured sequence. However apes KL and FK are - to our knowledge - the first attested non-humans to date to show isomorphisms, and both animals display identical results: in our experiment, each statistical hypothesis is either rejected or not identically for both individuals.

Cross-modal interactions can occur either at a decisional or at a perceptual level (Spence, 2011). In our experiment, choice of correct visual stimuli was significantly delayed by an incongruent auditory prime, but there was no association between sound played and chosen image (non-significant Fisher’s exact test). This suggests that auditory priming might have affected perception of visual structures rather than chimpanzees’ decision and choice of the structures.

Our results, which should be complemented by testing additional individuals and species and employ a more balanced experimental design, indirectly suggest that cross-modal isomorphisms might have been present in humans’ and chimpanzees’ last common ancestor. An open question is why humans and chimpanzees exhibit cross-modal isomorphisms, and whether these are based on shared, homologous neural mechanisms (Wilson, Marslen-Wilson, & Petkov, 2017). The “leakage” hypothesis suggests that cortical areas influence each other by proximity, facilitating for instance colour-number sequences mappings (Hubbard and Ramachandran, 2003, Ramachandran and Hubbard, 2001). Hence, synaesthesia and cross-modal associations might be quite common in chimpanzees because, unlike in humans, natural selection has not pruned this cross-cortical leakage (Humphrey, 2012). To address alternative hypotheses on the evolutionary function of cognitive isomorphisms, future work should test additional individuals in appropriate setups (Claidiére et al., 2014, Fagot and Cook, 2006, Fagot et al., 2013, Rey et al., 2012), and compare species with different degrees of sociality (Bergman et al., 2003, Dahl and Adachi, 2013, Seyfarth and Cheney, 2009). Combining the presented stimulus-interference task with RMTS tasks may provide a powerful methodological battery to tackle questions on evolutionary, functional, mechanistic, and developmental aspects of (cross-modal) analogical reasoning. Stimulus-interference paradigms allow testing for spontaneous cross modal processing of structural analogies, invaluable when looking at the ontogeny of analogy for instance. RMTS tasks on the other hand can be designed to test the degree and characteristics of analogy formation, crucial for questions about mechanism and function of analogical inferences.

Moreover, future animal experiments could be designed to test two alternative hypotheses on how isomorphisms are cognitively processed (Altmann et al., 1995): (1) regularities are represented in a domain- or modality-independent way; (2) regularities are stored in one specific modality, and a domain-independent (analogy-like) process is used to map them to other modalities. For instance, animals could be trained on *acoustic* patterns, testing if visual priming facilitates auditory discrimination, in order to assess whether the unidirectional cross-modal transfer we observe here is, in fact, bidirectional. This testing procedure would also provide a better control condition than the one we have used in our experiment, where we have shown that incorrect trials are not affected by acoustic priming.

Human language and cognition do not appear essential to map abstract structures between modalities; cross-modal ability might instead predate human linguistic abilities (Cuskley and Kirby, 2013, Ghazanfar and Takahashi, 2014, Luo et al., 2010, Simner et al., 2010); for a recent perspective, see Nielsen & Rendall, in press. Our findings suggest that cross-modal encoding might be more common across animals than previously surmised, and introduce a new experimental paradigm to test this suggestion.

## Figures and Tables

**Table 1:** Median latency (number of trials in bold) for each combination of presented audio stimulus (rows) and chimpanzees’ choice of visual stimulus (columns). In parentheses, Spearman's rank correlation rho between latency and success in the pre-trial, including its significance level (* < 0.05; ** < 0.01).

	KL		FK	
	Visual symmetric	Visual edge	Visual symmetric	Visual edge
Audio symmetric	8.52 (.64^*^) **10**	17.59 (.72^*^) **9**	5.68 (.61^**^) **19**	7.79 (.15) **19**
Audio edge	14.27 (.61) **7**	13.33 (.81^**^) **12**	8.25 (.67^**^) **16**	8.01 (.29) **22**

## Appendix A Supplementary material

Supplementary Data S1 This is open data under the CC BY license (http://creativecommons.org/licenses/by/4.0/).

Supplementary Data S2 This is open data under the CC BY license (http://creativecommons.org/licenses/by/4.0/).
