# Supplementary material for: Chimpanzees process structural isomorphisms across sensory modalities
Source: Cognition. 2017 Apr;161:74–9. doi: 10.1016/j.cognition.2017.01.005 (PMC5348109; doi:10.1016/j.cognition.2017.01.005)
Supplement: Supplementary data 3 [file mmc3.doc]

**Chimpanzees process structural isomorphisms across sensory modalities**

Andrea Ravignani and Ruth Sonnweber

**Supplemental Experimental Procedures**

*Learning Dependencies in the Visual Domain*

Two chimpanzees (FK and KL) were trained to differentiate between (i) visual stimuli, which contained non-adjacent, feature-based dependencies (Figure S1) between the first and the last elements of a string of variable length and (ii) visual stimuli lacking such a dependency. Stings consisted of a series of horizontally concatenated geometrical shapes (individual shapes were surrounded by a black square frame, outer dimensions: 225x225 pixels) in different colours (Figure S1). These strings were up to seven elements long, but consisted of a minimum of three individual elements. Dependencies were feature-based in the sense that categories of elements (geometrical shapes) were matched at the dependent string positions (first and last element of a sequence had the same shape). Variation within element classes (colour of the geometrical shapes) was introduced as category-irrelevant information to aid the generalization process. Thus, consistently all correct stimuli had shape-dependencies between the first and the last elements, whereby the colour of the shapes of these elements could, but did not necessarily need to, be the same.

Chimpanzees were trained and tested using a two-alternative-forced-choice (2-AFC) task on a touch-sensitive screen (15 in. Elo Touch Systems, Carroll Touch Technology) connected to an Apple Mini Mac computer. Stimuli design, control of experiments, and data logging was managed by custom written Python code (www.python.com). For a more detailed description of the setup, visual stimuli, and training/testing regime see . The two individuals who participated in this study successfully learned the feature-based dependency rule and generalized it over novel patterns. Thus, both chimpanzees were shown to be capable of processing non-adjacent, feature-based dependencies in strings of visual shapes .


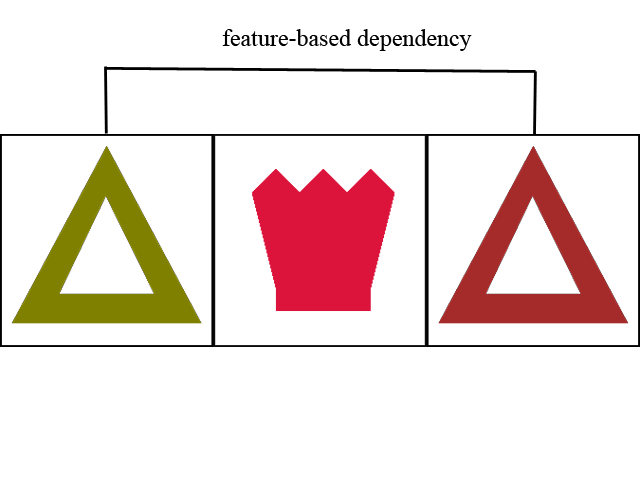


Figure S1: Example of a visual stimulus containing a feature-based dependency. The first and the last element (dependent elements) of the string are matched in shapes (dependency feature; both elements belong to the same category, in this case triangles) but not in colour (variation within the category). The middle element differs in shape from the dependency elements.

*Testing Cross-Modal Processing*

The two chimpanzees had been trained extensively for symmetric strings and successfully tested for generalizations of the rule to novel visual patterns; hence, they should choose symmetric stimuli over patterns consisting of edge-repetitions of same elements (termed edge stimulus hereafter), irrespective of the structure of the sound sequence played previously. However, if the structural regularity learned in the visual domain is encoded on a cross modal cognitive level an effect of the acoustic stimulus type will be expected: acoustic strings isomorphic/consistent with the symmetric structure of the correct visual stimulus should not alter the chimpanzees’ behaviour. When an acoustic stimulus with an edge structure interferes (as it is non-isomorphic/inconsistent with the visual rule learned) with processing the symmetry rule in the visual domain, a delay in choosing the symmetric visual stimulus should occur. Such a delay would be expected only if the structural regularity was encoded on a modality-general level.

To avoid a drop of motivation during the experiment each test trial was preceded by presentation of a set of familiar stimuli (chimpanzees had been trained previously to choose a red over a green circle, Figure S2, panel 1). In this pre-trial, responses were fed-back with two different types of sounds for correct (clicker sound familiar to the chimpanzees as a positive reinforcement sound) and incorrect (unappealing signal consisting of short, irregular sounds) answers. Additionally, a red penalty screen was displayed (for 3 seconds) after incorrect responses, or a highly preferred food reward (e.g., grapes, blueberries, peanuts, raisins, or pieces of dates) was given for correct choices. (This procedure was analogous to the testing regime used in the previous visual pattern learning experiment ).

Figure S2: Schematic of the experimental procedure. The chimpanzees were presented with a screen depicting a red and a green circle (pre-trial, panel 1). Previously they had been trained to choose the red over the green stimulus. When an individual touched the red circle a clicker sound was played and a food reward given. If the individual touched the green circle an unappealing, irregular sound signal was played, a red penalty screen was displayed (for 3s), and no food reward was given. After the pre-trial, a screen with a red circle was shown to ensure that the animal was paying attention (panel 2). The moment the animal touched the circle a symmetric (isomorphic with the visual rule) or edge (non-isomorphic with the visual rule) acoustic stimulus was played. Consequently two visual stimuli (one symmetric and one edge, panel 3) were displayed and the chimpanzee could make its choice by touching either of them. After a one-second inter-trial interval another pre-trial screen was shown.

*Visual and Acoustic Patterns*

Table S1: Overview of acoustic-visual stimuli combinations. Half of the acoustic patterns (low-high-low or high-low-high sounds) matched the (previously learned) symmetric visual pattern, while the other half (low-low-high, high-high-low, low-high-high, high-low-low) followed the edge pattern of the negative visual stimuli.

| **isomorphic / non-isomorphic** | **number of stimuli** | **acoustic patterns** | **visual patterns** | |
| --- | --- | --- | --- | --- |
|  |  |  | **edge** | **symmetric** |
| non-isomorphic | 10 | LLH | AAX | AXA |
| non-isomorphic | 10 | HHL | AAX | AXA |
| isomorphic | 10 | LHL | AAX | AXA |
| isomorphic | 10 | HLH | AAX | AXA |
| non-isomorphic | 10 | LHH | XAA | AXA |
| non-isomorphic | 10 | HLL | XAA | AXA |
| isomorphic | 10 | LHL | XAA | AXA |
| isomorphic | 10 | HLH | XAA | AXA |

Statistical analyses were performed using SPSS 19. Unless stated otherwise, all p-values are exact and two-tailed.

**Supplemental Results**

Significant differences in response latencies were found between isomorphic and non-isomorphic conditions. Chimpanzee FK’s latency in choosing the symmetric visual stimulus was significantly longer when an non-isomorphic auditory stimulus was played than when the auditory stimulus had a symmetric (isomorphic) pattern (Mann-Whitney U test N=35, U(33)=80, W=270, Z=-2.384, p=0.017; mean ranks: (isomorphic) 14.21; (non-isomorphic) 22.50). When choosing the edge visual stimulus however, ape FK did not show any difference between patterns of auditory stimuli (Mann-Whitney U test N=41, U(39)=199, W=452, Z=-.261, p=0.806; mean ranks: (isomorphic) 21.53; (non-isomorphic) 20.55).

Chimpanzee KL presented a response pattern analogous to FK. Playing an non-isomorphic, auditory edge pattern increased KL’s latencies to choose the symmetric visual pattern (Mann-Whitney U test N=17, U(15)=10, W=65, Z=-2.440, p=0.014; mean ranks: (isomorphic) 6.50; (non-isomorphic) 12.57), while latencies did not differ between auditory conditions when the edge visual stimulus was chosen (Mann-Whitney U test N=21, U(19)=48, W=126, Z=-.426, p=0.702; mean ranks: (isomorphic) 11.67; (non-isomorphic) 10.50).

*Analyses without exclusions*

Chimpanzee FK was significantly slower in answering when a non-isomorphic auditory stimulus interfered (median latency=8.24 s) than when an isomorphic acoustic stimulus was played (median latency=5.68 s). Similarly, ape KL showed longer latencies in the non-isomorphic condition (median latency=14.27 s) than in the isomorphic condition (median latency=8.52 s) (Table S2).

Table S2: Results when last trial is included (results, conclusions, and interpretation remain unaltered from results with balanced trial data set)

| Ape | Response | N | df | U | W | Z | p | Mean rank  symmetric | Mean rank scrambled |
| --- | --- | --- | --- | --- | --- | --- | --- | --- | --- |
| F | Correct | 36 | 34 | 90 | 280 | -2.266 | .023 | 14.74 | 22.71 |
| F* | Incorrect | 41 | 39 | 199 | 452 | -0.261 | .806 | 21.53 | 20.55 |
| K* | Correct | 17 | 15 | 10 | 65 | -2.440 | .014 | 6.50 | 12.57 |
| K | Incorrect | 22 | 20 | 55 | 133 | -0.330 | .771 | 12.00 | 11.08 |

*Latencies to respond to visual stimuli without acoustic interference*

To assure that this result was not due to differences in response latencies to visual symmetric and edge stimuli, we compared latencies between correct and incorrect choices when presented with visual stimuli only (no acoustic stimuli played before presentation of a set of visual stimuli). All responses to three-element long (comparable to visual stimuli used in this experiment), reinforced stimuli presented during tests in were used for this analysis. We found no differences in latencies between correct (symmetric stimulus was chosen) and incorrect (edge stimulus was chosen) answers (Mann-Whitney U test; FK: N=108, U=891, W=4719, Z=-.175, p=0.861; mean ranks: (correct responses) 54.24; (incorrect responses) 55.57; KL: N=106, U=868, W=1193, Z=-1.075, p=0.282; mean ranks: (correct responses) 55.28; (incorrect responses) 47.72). Therefore we conclude that differences in latencies to respond in isomorphic and non-isomorphic audio-visual trials are due to a cross-modal interference of the acoustic sequence with the structure of the visual stimulus.

**Description of data sets**

The files ‘data_KL.csv’ and ‘data_FK.csv’ each contain the data obtained from an individual chimpanzee experiment. The meaning of each variable in the data set is as follows:

*SBJname*: name of the individual chimpanzee;

*sound*: sound heard (0= edge triplet, 1= symmetric triplet);

*response*: visual stimulus chosen (0=edge triplet, 1=symmetric triplet);

*latency*: latency to respond in seconds;

*serialID*: combination of the auditory presentation and visual choice as defined by variables sound and response (i.e. simple recoding of these variables in 4 classes);

*pre-trial*: performance in the pre-trial preceding the current experimental trial (0=incorrect, 1=correct).

**Supplemental References**

Sonnweber, R., Ravignani, A., & Fitch, W. T. (2015). Non-adjacent visual dependency learning in chimpanzees*. Anim C*og, 1-13.
